# Ecological indicators for qualitative assessment of Ojarud River: A case study

**DOI:** 10.1002/ece3.10310

**Published:** 2023-07-17

**Authors:** Aydin Mobasher, Abolfazl Bayrami, Ehsan Asadi‐Sharif, Shima Rahim Pouran

**Affiliations:** ^1^ Department of Biology, Faculty of Science University of Mohaghegh Ardabili Ardabil Iran; ^2^ Department of Soil and Water Research Gilan Agricultural and Natural Resources Research and Education Center, AREEO Rasht Iran; ^3^ Department of Environmental and Occupational Health, Social Determinants of Health Research Center Ardabil University of Medical Sciences Ardabil Iran

**Keywords:** biotic indices, heavy metal, macro‐invertebrates, Ojarud River

## Abstract

Today, the application of ecological indicators based on organisms has replaced traditional saprobic approaches for assessment of the quality of rivers impaired due to organic pollution and some other environmental disturbances. This study aimed to weigh the quality of the Ojarud River in Ardabil, Iran, applying biological and physiological indices of macro‐invertebrates. A total of 12,524 samplings were fulfilled at four stations (S1, S2, S3, S4) from the headstream to downstream by a Surber sampler (30 × 30 cm^2^) from June/2020 to April/2021. All year round, the highest frequent families were Chironomidae (2658), Simuliidae (1025) from Diptera and Caenidae (1855), and Baetidae (724) from Ephemeroptera. The diversity pattern was analyzed by PAST software, and Primer 7 (BIO‐ENV analysis) was utilized to understand what factor has the most impact on the distribution of macro‐invertebrates. The least similarity of S4 to other stations was recognized by Cluster analysis. As per the ANOSIM (analysis of similarities), a statistically significant difference in the macroinvertebrates' frequency was established between S3 and other stations (*p* = .0001, *r* = .63). Moreover, the relationship between heavy metals and macro‐invertebrate showed that the three families of Simuliidae, Gomphidae, and Caenidae had a positive correlation with the concentrations of heavy metals in the sediment. As per the Ephemeroptera, Plecoptera and Trichoptera index, the water quality was placed in the “excellent” class, but the Biological Monitoring Working Party and Hilsenhoff Family Biotic Index indices scored the water quality “good” class at S1 and the “poor” class at S3. Based on the results of this study, the use of physicochemical and hydro‐morphological indicators can support the biological indicators but cannot replace them. In addition, careful evaluation of biological indicators is required to develop conservation strategies.

## Graphical abstract

The results of this study exposed a sizable alteration in the diversity and abundance of macro‐invertebrate from the sensitive families to resistant and tolerant taxa under the contamination stress resulting from the point source pollution in the Ojarud River. The results of this study, yet again, verified the simultaneous applications of various indices for a more effective assessment of the health of a stream.

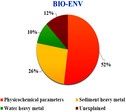

## 1 INTRODUCTION

Out of the 17 Sustainable Development Goals (SDGs), “clean water and sanitation” is listed as the sixth goal of the United Nations (UN) goal to “Ensure availability and sustainable management of water and sanitation for all” (United Nations, 2022). As per the UN report, water‐based ecosystems (mainly lakes and rivers) are swiftly being debased worldwide. Apart from the natural processes such as hydrological run‐off, leaching from the soil, and rock weathering, anthropogenic activities have led to a vast discharge of industrial and domestic wastewater, and agricultural pollutants into natural water‐bodies and intensively contributed to the poor water quality and loss of biodiversity of receiving aquatic environment.

Biological indicators have become an acceptable alternative to traditional approaches such as saprobic indices for assessment of the health status of river ecosystems (Ibáñez et al., 2010). The initial studies on applying biological indicators were carried out in Europe and the United States (Persoone & De Pauw, 1979). However, monitoring of river health via biological indicators becomes more prevalent in recent years in developing countries such as Iran (Aazami et al., 2015; Asadi Sharif et al., 2020; Shokri et al., 2014). The health condition of the river is usually assessed via three categories of organisms:

1. Benthic diatoms (i.e., phytobenthos) to detect eutrophication and acidification status (Eveleens Maarse et al., 2020; Masouras et al., 2021).
2. Fish species to detect habitat modifications and flow alterations (Jumani et al., 2018).
3. Macro‐invertebrates to monitor the impact of acidification, hydro‐morphological changes, and organic pollution on river health (Munyika et al., 2014; Rico‐Sánchez et al., 2022).

The underlining reasons for the popularity of the macroinvertebrate‐based bioassessment of river health could be its continuous monitoring of water quality in contrast to one‐time sampling for chemical experiments, the abundance of taxa, their sedentary lifestyle and long lifetime, facial sampling and identification, presence in all areas of the river (crenal, rhithral, and potamal zones), distinct responses of each taxon to physicochemical pollutions, for example, acidification, eutrophication, and organic enrichment, and anthropogenic activities for example, river regulation, impoundment, and canalization, as well as playing a key role in effective river food web along with river continuum (Alba‐Tercedor, 2006; Ficsór & Csabai, 2021; Sumudumali & Jayawardana, 2021).

The presence of heavy metals in water and/or sediment of rivers and their impacts on living organisms is a matter of serious concern. Natural processes such as atmospheric precipitation, geological weathering, erosion, and bioturbation and/or anthropogenic activities, for example, industrial discharge, agricultural and urban activities mining, and transportation are among the main origins of heavy metals (Bradl, 2005). Unlike most pollutants, heavy metals are not bio‐degradable and can be concentrated throughout the food chain and exert toxic effects on human health and the environment (Ali et al., 2019). Since macro‐invertebrates engage in nutrient recycling and supply food to higher tropical levels, the increase in the concentration of heavy metals in rivers can lead to their accumulation to varying degrees in the food chain and disruption of its function.

The present study aimed to assess the health status of the Ojarud River in northwest of Iran. This river is not only the main source of water for agriculture in the area but also could offer opportunities to investigate some key factors as the main objectives of this study: (i) The effects of the anthropogenic stress on the macrobenthos diversity. It was especially important to know the effects of the sewage effluent, which was combined with a lot of foam in the third station (Figure S1) on the distribution and diversity of the macrobenthic diversity of the studied river. (ii) The effect of the seasonal changes on the distribution of benthic invertebrates and also the replacement of pollution‐sensitive species with pollution‐resistant species. (iii) Validation of a number of well‐performing biotic indexes (EPT, HFBI, and BMWP) and to know whether these biological indicators are effective to perform under the corresponding flora and fauna of the studied river. (iv) Assessment of the impact of heavy metals on benthic invertebrates as biological indicators. This has been given less attention in the biological monitoring of rivers as a strategic source of agricultural irrigation, and most studies only use biological indicators of benthic invertebrates for monitoring rivers. As reported earlier, the combination of these two methods has provided more accurate and reliable results (Kaboré et al., 2022; Nahli et al., 2022). Accordingly, the study was designed to investigate the physicochemical aspects of the Ojarud River; identify its macro‐invertebrates at the family level; assess the effects of the wastewater treatment plant as the point source of pollution on the health condition of the river; and apply PRIMER software to detect the most effective parameter on the spatial distribution of macro‐invertebrates.

## 2 MATERIALS AND METHODS

### 2.1 Study region

**FIGURE 1:**
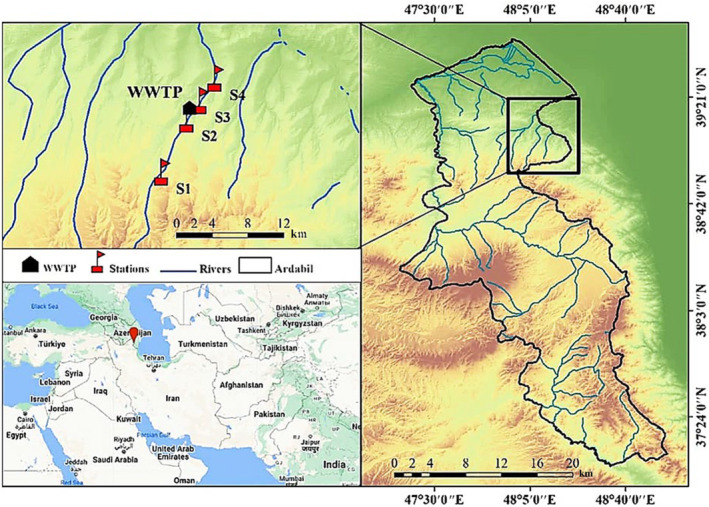
The locations of the sampling stations on the Ojarud River (blue line), Ardabil, Northwest of Iran, and the WWTP (Waste Water Treatment Plant; between S2 and S3).

Ojarud River, locally known as Germi Chai, located about 110 km from Ardabil City, Iran, originates from the Toulon River and drains into the Caspian Sea. The length of the Ojarud River is over 55 km and passes along a wastewater treatment plant where the ecosystem of this area is under physical and biological disturbances due to this source of pollution. Four sites were chosen along the longitudinal Germi Chai River gradient (illustrated in Figure 1) and samplings were carried from June 2020 to September 2021, starting in summer, and continuing in autumn, winter, and spring. The selection of sampling stations was per the pollution sources of the river (point and non‐point):

S1: The first station was selected at upstream of the river, where the aquatic plants grow nearby water all year round.
S2: The second station was selected before the wastewater treatment plant. In this station also aquatic plants and freshwater algae were seen along the river.
S3: The third station was selected about 1 km after the wastewater treatment plant. The contamination caused by the sewage effluent gave rise to an unpleasant odor due to the formation of toxic foam.
S4: The fourth station was selected in the downstream region of the river where agricultural activities were developed surrounding the station in recent times. The geographical coordinates and characteristics of the selected sampling sites are detailed in Table 1.

**TABLE 1:** Geographical coordinates and characteristics of the sampling stations.

Station no.	Coordinates	Altitude (FT)	Flow rate (m/s)	Vegetation	Substrate
1	38°34′6.89″; 48°34′6.89″	1356	0.59	Pasture	Large cobbles‐clay and silt
2	39°3′20.57″; 48°6′2.8″	700	0.59	Shrub‐lands under agriculture	Sand and gravel
3	39°4′25.80″; 48°7′10.59″	628	0.54	Shrub‐lands under agriculture	Sand and gravel
4	39°5′42.95″; 48°8′27.79″	545	0.41	Shrub‐lands under agriculture	Sand and gravel

### 2.2 Sampling

For mesohabitats (edge and riffle), the Surber sampler (area: 0.09 m^2^, dimensions: 0.3 m × 0.3 m) was used for macro‐invertebrates sampling. Standard protocols were followed and three‐time repeated for the bioassessment of the river (U.S.Environmental‐Protection‐Agency, 2015). At the sites, the samplings were sieved using mesh screens (500 mm) and collected in plastic jars. The formalin (5%) preservation of the macro‐invertebrates was carried out until the counting and laboratory analyses (APHA, 1999). The river water and sediment macro‐invertebrates were identified at the family level using a stereo microscope (Oscoz et al., 2011).

The in‐situ analysis of water's physical properties was carried out using a portable multi‐parameter analyzer (WTW) to determine the pH, temperature (°C), dissolved oxygen (mg/L), and electrical conductivity (μs/cm). The phosphate and nitrate concentrations and biological oxygen demand (BOD) were determined using the American Public Health Association procedures (APHA, 1999). An atomic absorption spectrophotometer (Varian SpectraAA 220 FS, USA) was employed to quantify the concentrations of heavy metals (cadmium and lead) in water, sediment, and selected macro‐invertebrates.

### 2.3 Biotic indices and diversity

Since the biological communities respond to environmental stress via different adaptive strategies, the biological indicators vary with respect to the ecosystem type. Today, there are numerous biotic indices proposed for surface water assessment such as River Invertebrate Prediction and Classification Scheme (RIVPACS; Wright & Ryan, 2016), Stream Invertebrate Grade Number Average Level (SIGNAL; Chessman, 2003), and Australian River Assessment System (AUSRIVAS; Sudaryanti et al., 2001). Three water quality biotic indices, including the index of Ephemeroptera, Plecoptera, and Trichoptera (EPT; Akamagwuna & Odume, 2020), Biological Monitoring Work Party Scoring System (BMWP; Hawkes, 1998), and Hilsenhoff Family Biotic Index (HFBI; Hilsenhoff, 1988), and three taxonomic biodiversity indices including Shannon‐Wiener diversity (*H*), Simpson (1 − *D*) and Dominance (*D*) were applied in this study.

### 2.4 Data analysis

The statistical analysis of the data was performed using PAST statistical software (version 3.25) wherein the *p*‐value was calculated at the level of significance of 5%. The data normality was set by Shapiro–Wilk test. The parameter which had the highest impact on the distribution of macro‐invertebrates was determined via Primer 7 (BIO‐ENV analysis). The differences among the stations were investigated by several multivariate techniques (ANOSIM, SIMPER, and CLUSTER). ANOSIM (one‐factor analysis of similarities) was used to compare the structure of the macroinvertebrates' communities in varying seasons. In addition, the square root of the Bray–Curtis similarity (SIMPER test) was used to determine the average contribution rate of each macroinvertebrate species (Auer et al., 2014). The group‐averaging cluster analysis was performed based on the Bray–Curtis similarity. Shannon‐Winner's diversity index (*Ĥ*), Dominance (*D*), and Simpson (1 − *D*) were applied as the univariate diversity indices. Finally, the correlations among the macroinvertebrates, environmental variables, and leached heavy metals were established using canonical correspondence analysis (CCA‐CANOCO 5; Ter Braak & Smilauer, 2013).

## 3 RESULTS

### 3.1 Macroinvertebrates

In the course of this project, 12,524 samples were collected and identified from four stations, which belonged to 10 orders and 17 families (Table 2). The number of samples during the f seasons was highest in the order of spring (2974) > autumn (2444) > summer (2214) > winter (1892), respectively. Ephemeroptera and Diptera were the most frequent orders and Pulmonata, Lepidoptera, and Coleoptera were among the least frequent orders. As per seasonal data, the highest biomass of macro‐invertebrates was recorded in summer (0.789 g/m^2^) followed by spring (0.653 g/m^2^), autumn (0.591 g/m^2^), and winter (0.475 g/m^2^). As per the stations, the highest biomass was observed in station 4 in summer (0.289 g/m^2^) and the lowest biomass was observed in station 3 in winter (0.089 g/m^2^). The reason for the high biomass in S4 could be related to the large number of the Gomphidae family in S4. It is noteworthy to mention that the members of the Gomphidae family are among the largest and heaviest macro‐invertebrates with a length of about 16 mm and a relatively higher dry weight of approximately 0.018 g.

**TABLE 2:** A list of macro‐invertebrates recorded in four stations on the Ojarud River.

Phylum	Class	Order	Family	S1	S2	S3	S4
Arthropoda	Insecta	Plecoptera	Perlidae	*			*
Arthropoda	Insecta	Ephemeroptera	Baetidae	*	*	*	*
			Caenidae	*	*	*	*
			Heptageniidae	*			
Arthropoda	Insecta	Trichoptera	Hydropsychidae	*	*		*
			Limnephilidae	*			
Arthropoda	Insecta	Diptera	Chironomidae	*	*	*	*
			Simuliidae	*	*	*	*
			Tabanidae	*	*		
			Tipulidae	*			
Arthropoda	Insecta	Odonata	Gomphidae	*	*	*	*
			Coenagrionidae	*			*
Arthropoda	Insecta	Coleoptera	Dytiscidae				*
Arthropoda	Insecta	Lepidoptera	Pyralidae		*		
Mollusca	Gastropoda	Pulmonata	Physidae	*	*	*	*
Annelida	Clitellata	Hirudinida	Glossiphoniidae	*	*	*	*
Annelida	Clitellata	Oligochaeta	Tubificidae	*	*	*	*

From the seasonal data, it was found that the Ephemeroptera order showed the highest frequency at S2 in the autumn and Diptera was the most abundant order at S3 in summer, which showed a statistically significant difference with the results in other seasons (*p* < .05). The frequency of the families was also assessed in each station. The most frequent families in S1 were Baetidae and Caenidae families (average number of 64 and 46), in S2: Caenidae and Baetidae with an average number of 113 and 53, in S3 (after the point source of pollution): Chironomidae and Simuliidae families with average number of 456 and 147, and finally, in S4: Simuliidea and Chironomidae families with average number of 208 and 198. The images of a number of identifies families are provided in Figure S2.

### 3.2 Physical–chemical properties of the Ojarud River

The physicochemical properties of the river were determined in each season through both in‐situ and ex‐situ analyses. The data on the physical and chemical variables are given in Table 3. The maximum and minimum temperatures were, respectively, recorded in S3 (30.5°C) in summer and S1 (11°C) in winter. Although there was no statistically significant difference in temperature among the stations (*p* > .05), the inter‐seasonal comparisons revealed a significant difference in temperature between winter with other seasons (*p* < .05). The maximum and minimum dissolved oxygen (DO) were, respectively, found in S1 (9.2 mg/L) in winter and S3 (7.6 mg/L) in autumn. In contrast, the highest concentration of biochemical oxygen demand (BOD) was recorded in S3 (18 mg/L) in summer, while it was the least in S1 (0.9 mg/L) in winter. As per the decreased water flow and increased point source pollution intensity, a rise in BOD was observed in S3 in summer. The concentration of ${\mathrm{NO}}_{3}^{-}$ varied between 63.278 mg/L (in S2 in winter) and 14.1 mg/L (in S1 in autumn). In contrast, the highest and lowest concentrations of ${\mathrm{PO}}_{4}^{3-}$ were, respectively, recorded at S2 and S3 during the summer and spring (6.13 mg/L), and at S2 in autumn (0.25 mg/L).

**TABLE 3:** Characteristics of the sampling sites in Ojarud River.

Season	Parameter	Mean ± SD	*p* Value
Winter	${\mathrm{NO}}_{3}^{-}$	42.66 ± 18.17	<.05
	${\mathrm{PO}}_{4}^{3-}$	3.37 ± 1.36	<.05
	DO	8.28 ± 0.70	<.05
	BOD	3.42 ± 3.39	<.05
	pH	8.15 ± 0.24	<.05
	TEM	14.65 ± 2.36	<.05
	FLOW	0.595 ± 0.047	<.05
Autumn	${\mathrm{NO}}_{3}^{-}$	38.30 ± 15.96	<.05
	${\mathrm{PO}}_{4}^{3-}$	2.50 ± 1.87	<.05
	DO	8.39 ± 0.541	<.05
	BOD	3.59 ± 4.10	<.05
	pH	8.18 ± 0.31	<.05
	TEM	26.11 ± 1.82	<.05
	FLOW	0.595 ± 0.047	<.05
Spring	${\mathrm{NO}}_{3}^{-}$	23.70 ± 22.96	<.05
	${\mathrm{PO}}_{4}^{3-}$	3.04 ± 0.22	<.05
	DO	8.28 ± 0.38	<.05
	BOD	4.67 ± 4.56	<.05
	pH	8.11 ± 0.28	<.05
	TEM	19.37 ± 3.18	<.05
	FLOW	0.54 ± 0.10	<.05
Summer	${\mathrm{NO}}_{3}^{-}$	21.29 ± 5.38	<.05
	${\mathrm{PO}}_{4}^{3-}$	4.00 ± 2.15	<.05
	DO	8.31 ± 0.42	<.05
	BOD	6.64 ± 6.66	<.05
	pH	8.03 ± 0.32	<.05
	TEM	26.69 ± 2.08	<.05
	FLOW	0.41 ± 0.055	<.05

### 3.3 Heavy metals

**FIGURE 2:**
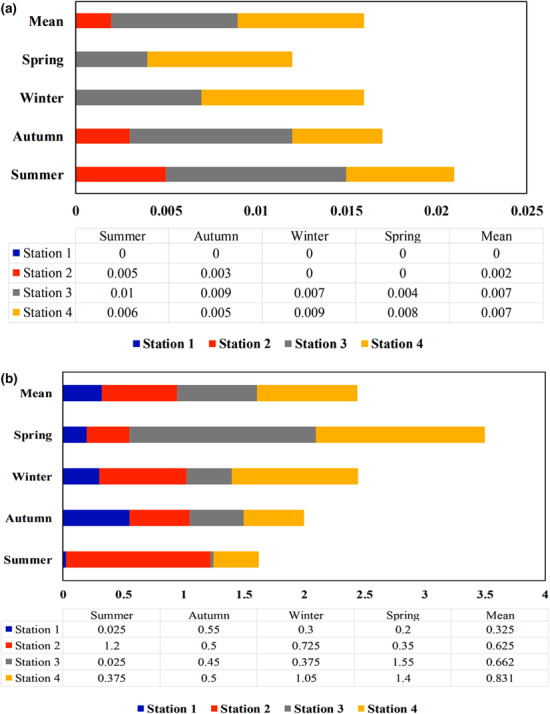
The concentration of cadmium in (a) water (mg/L) and (b) sediment (mg/kg).

**FIGURE 3:**
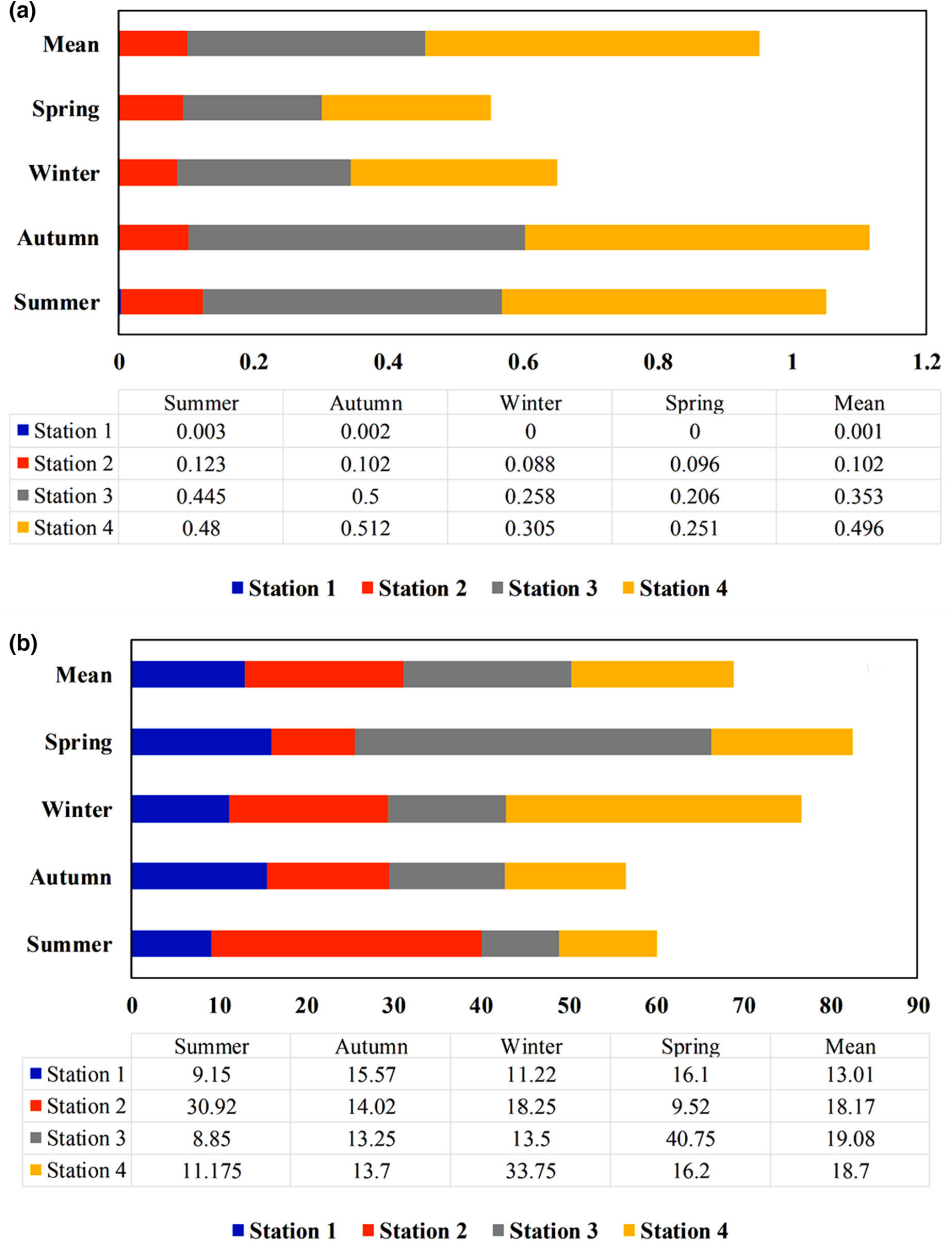
The concentration of lead in (a) water (mg/L) and (b) sediment (mg/kg).

To assess the heavy metal contamination status of the river, the concentrations of cadmium and lead were measured in water, sediment, and two macro‐invertebrates in different seasons. In the upstream region, the average river cadmium was lower than that of the aquatic biological and surface water standards, while the concentration was significantly higher downstream of the river. In contrast, the concentration of lead in river water of S2, S3, and S4 was greater than that of the international standards. In contrast, the average concentrations of cadmium and lead in sediment were higher in the downstream stations than in the upstream region, however, it was still below the international standards of freshwater sediments (Figures 2 and 3). In order to evaluate the bioaccumulation potential of Cd and Pb in macro‐invertebrates, Simuliidae and Gomphidae families were selected as the most abundant family owning the highest total length compared to other families. The concentrations of Cd and Pb were 0.059 and 1.392 mg/kg in Simuliidae and 0.031 and 0.773 mg/kg in Gamphidae, respectively.

### 3.4 BIO‐ENV analysis

**FIGURE 4:**
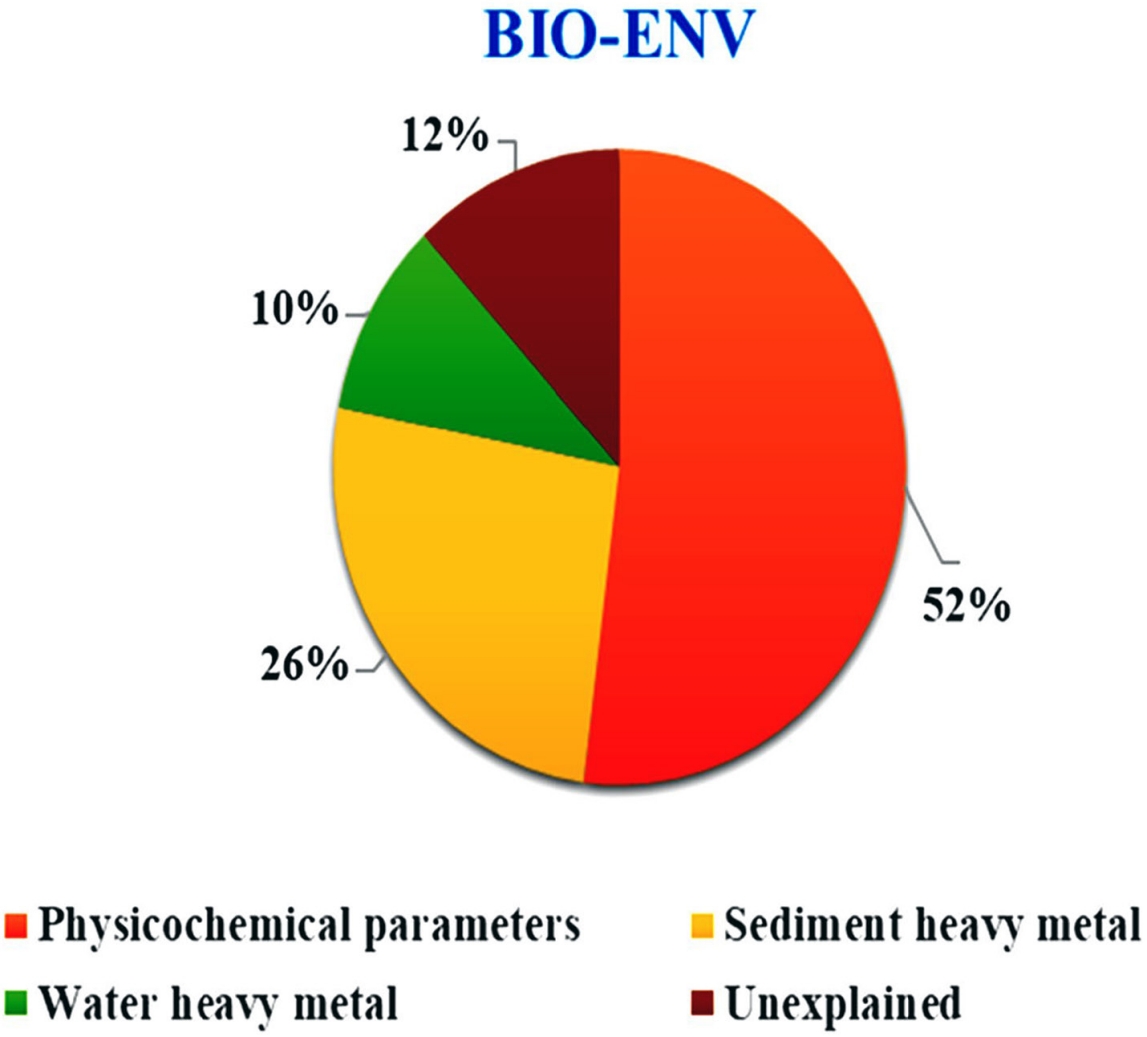
The output of BIO‐ENV analysis.

Primer 7 (BIO‐ENV analysis) was employed to understand what factor has the greatest impact on the distribution of macro‐invertebrates. The results of BIO‐ENV statistical analysis showed that the most influential factors in the distribution of macro‐invertebrates were physicochemical parameters (52%), sediment heavy metals (26%), and finally water heavy metals (10%), respectively (Figure 4). A comparison between water and sediment heavy metals showed that heavy metals in the sediment had a greater impact on the distribution of macro‐invertebrates.

### 3.5 Multivariate analysis and biotic indices

**FIGURE 5:**
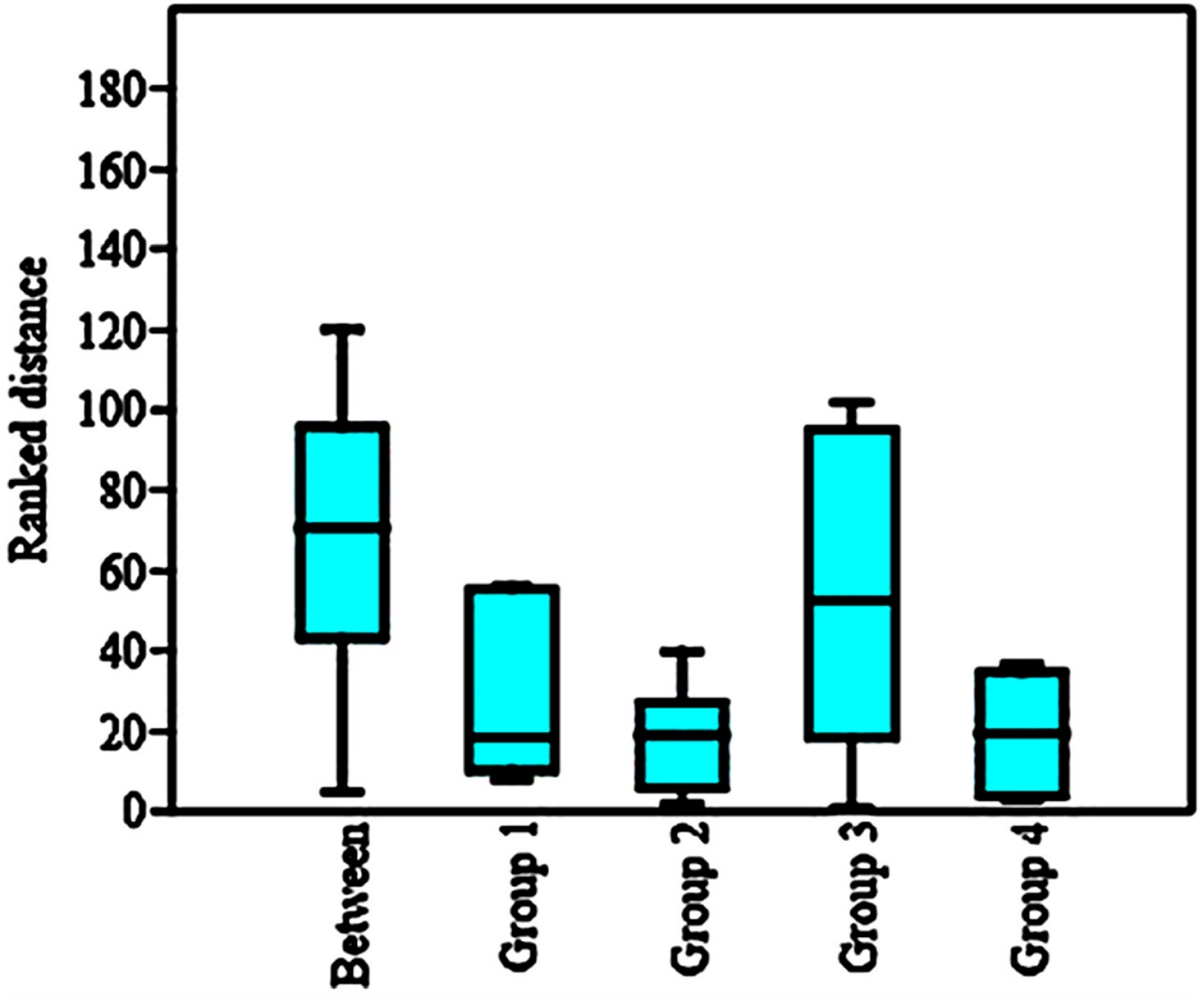
Analysis of similarities based on the Bray–Curtis dissimilarity index.

Analysis of similarities (ANOSIM) was used to compute the difference between the macro‐invertebrates' communities among S1 to S4 based on Bray–Curtis dissimilarity index (Clarke, 1993). As per the results, S3 had a statistically significant difference with other stations in terms of the frequency of macro‐invertebrate (*p* = .0001, *r* = .63; Figure 5). In addition, the SIMPER procedure (Clarke, 1993) was used to assess the average contribution of taxa to the dissimilarity (in percent) among the groups of the samples in four seasons. The results of SIMPER analysis showed that the Chironomidae and Simuliidae families were the major taxa with the highest contribution to the differences among family groups. The community dissimilarity indices are indicated in Table 4.

**TABLE 4:** Results of similarity percentages analysis (SIMPER) by the Bray–Curtis method at four sampling sites.

Season	Average dissimilarity (%)				
	Station	S1	S2	S3	S4
Summer	S1		35.24	84.39	51.92
	S2	35.24		67.21	34.33
	S3	84.39	67.21		57.88
	S4	51.92	34.33	57.88	
Autumn	S1		51.85	78.13	79.68
	S2	51.85		74.39	77.71
	S3	78.13	74.39		81.23
	S4	79.68	77.71	81.23	
Winter	S1		57.83	73.64	70.25
	S2	57.83		60.32	47.45
	S3	73.64	60.32		68.9
	S4	70.25	47.45	68.9	
Spring	S1		37.14	81.82	69.57
	S2	37.14		45.45	44.5
	S3	81.82	45.45		55.5
	S4	69.57	44.5	55.5	

**FIGURE 6:**
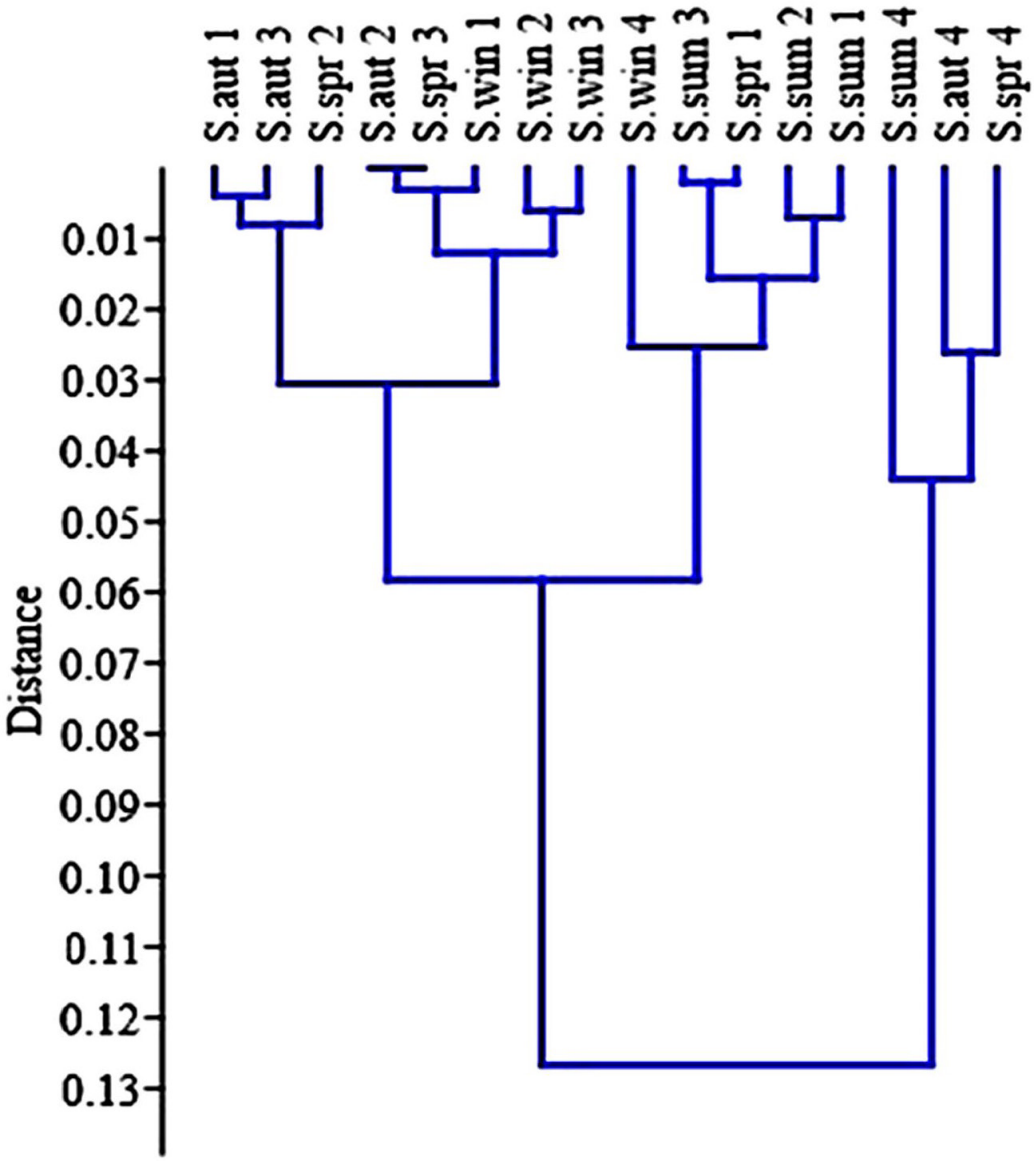
Cluster analysis of the macroinvertebrates' community collected in Ojarud River during four seasons in 2020–2021.

**FIGURE 7:**
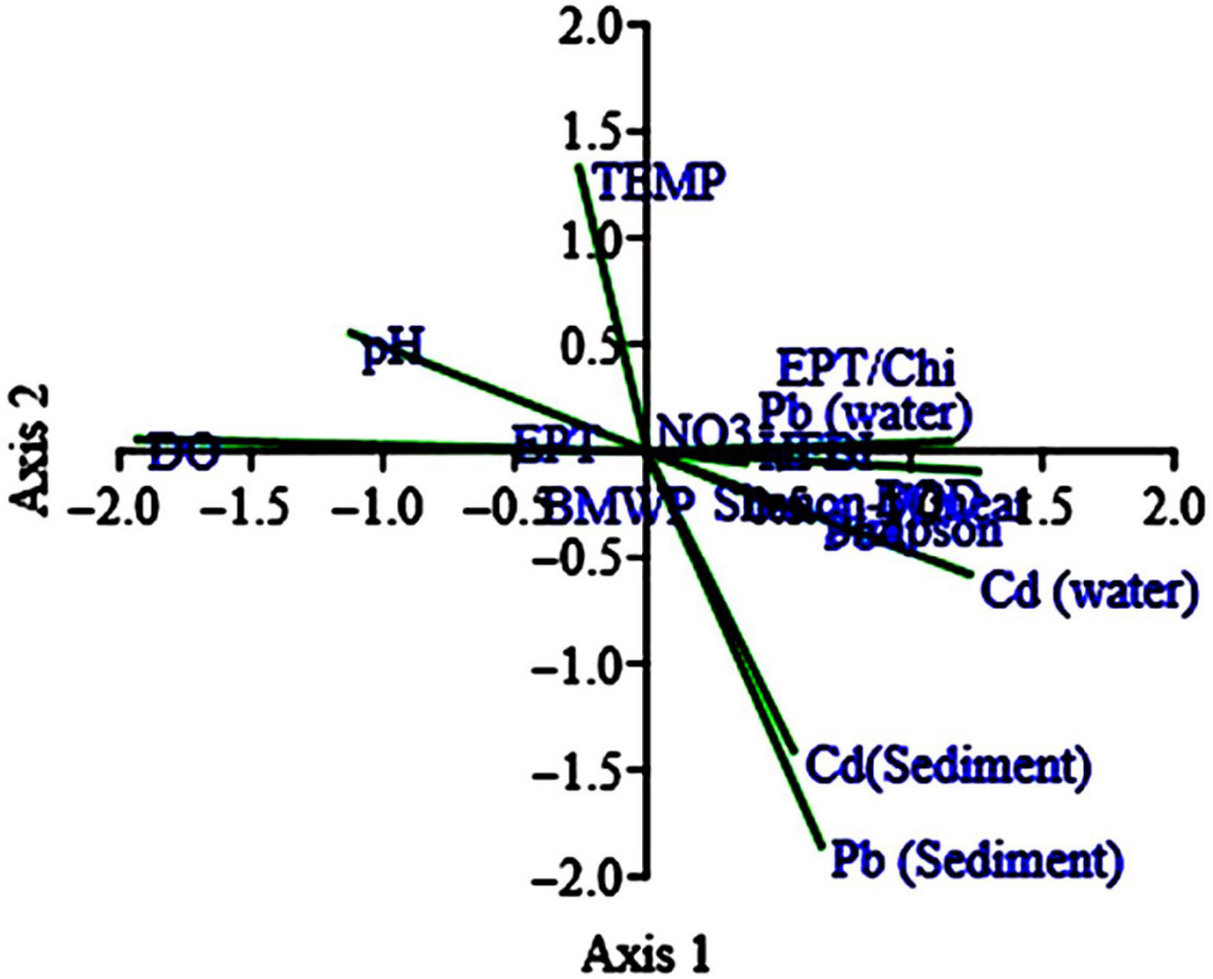
Canonical correspondence analysis ordination diagrams of biotic indices and environmental factors in Ojarud River.

The classical hierarchical cluster analysis based on a Bray–Curtis similarity index was used to compare the biomass similarity at four stations during all seasons. The results of this analysis revealed that S4 had the least similarity (23%) to other stations in three seasons (spring, summer, and autumn; Figure 6). Moreover, the relationships among the environmental factors, heavy metals, and biotic indices were assessed by canonical correspondence analysis (CCA). As per the CCA analysis, two biotic indices (BMWP, HFBI) and biodiversity indices (Simpson and Shannon‐Wiener) showed a positive correlation with heavy metals. However, the EPT biotic index showed a positive correlation with temperature, dissolved oxygen, and pH and a negative correlation with heavy metals (Figure 7). On the contrary, the study on the relationship between heavy metals and macro‐invertebrate revealed that three families (Simuliidae, Gomphidae, and Caenidae) had positive correlations with sediment heavy metals. The first two axes of CCA demonstrated 61.48% variance where CCA2 accounted for 24.11% variance with eigenvalues of 0.013.

**FIGURE 8:**
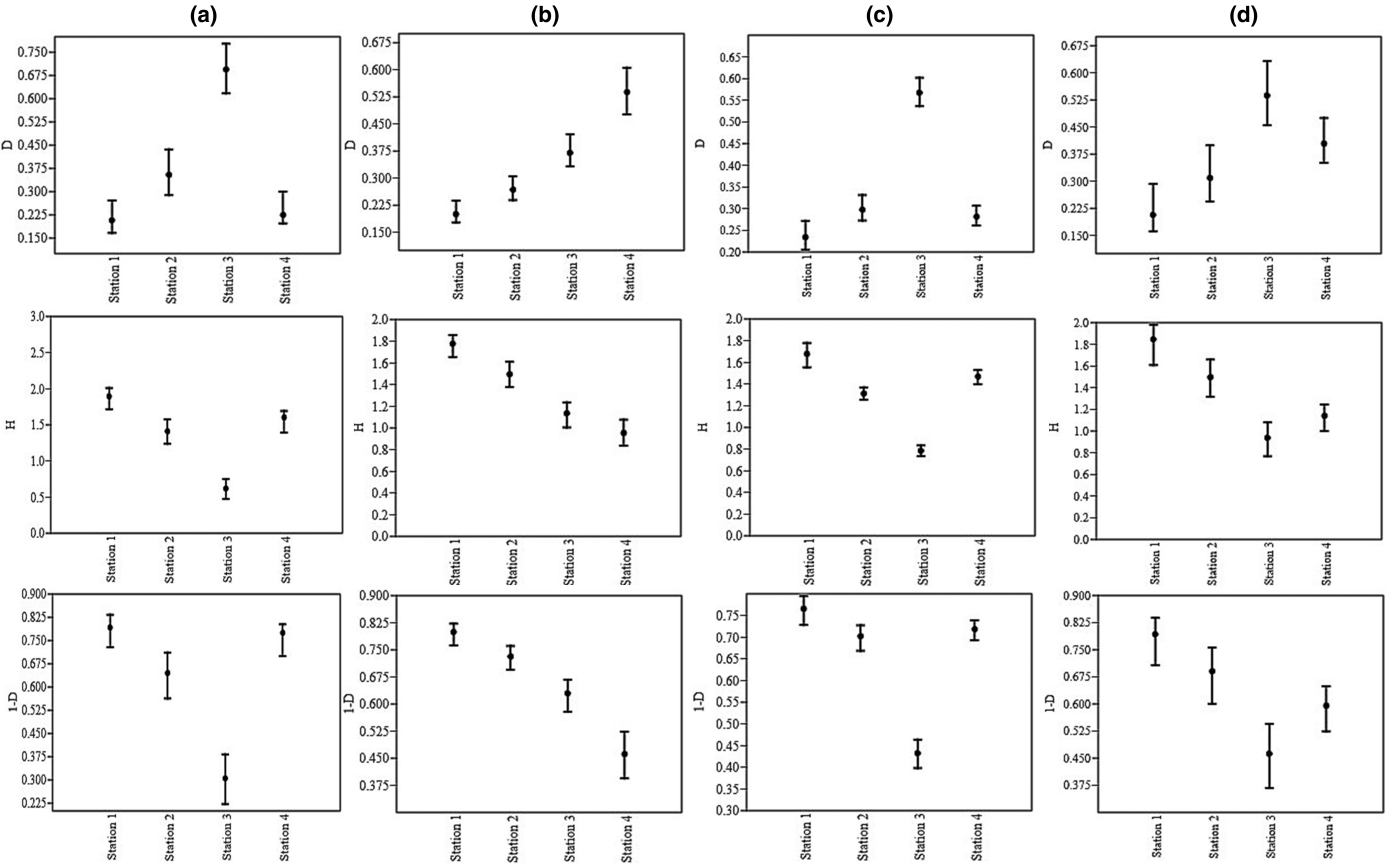
Taxonomic diversity (Dominance [*D*], Shannon‐Winner [*H*], and Simpson [1 – *D*]); (a) Autumn, (b) Spring, (c) Summer and (d) Winter at the four sampling sites in Ojarud River.

The results of Dominance (*D*), Shannon‐Winner diversity (*H*), and Simpson (1 − *D*) indices are presented in Figure 8. The Shannon‐Winner index (*H*) showed maximum values in S1 in winter (1.9) and minimum values in S3 in autumn (0.5). The Simpson index (1 − *D*) showed maximum values in S1 in autumn (1.9) while minimum values in S3 in summer (0.4). On the contrary, the Dominance diversity index (*D*) showed significant variations among the studied stations being highest in S3 in autumn (0.67) and lowest in S1 in winter (0.16).

The mean values of HFBI ranged from 4.53 to 7.56. The lowest value was at S1 in the winter and the highest value was at S3 in the winter (Table 5). The differences among sampling sites were significant (*p* < .05). Mean values for the BMWP index varied between 19 and 66.33. The differences among the mean values of BMWP were significant (*p* < .05) with the lowest and highest values at S3 in spring and S1 in summer, respectively. The mean values of the EPT (Ephemeroptera, Plecoptera, and Trichoptera) index were recorded from 25 to 325. The lowest value was at S3 in winter and the highest value was at S4 in summer.

**TABLE 5:** Mean ± SE of macro‐invertebrate metrics per site and season and summary of the health status of Ojarud River using various indices over a year.

Season	Station	EPT	Class	HFBI	Class	BMWP	Class
Summer	S1	164.33 ± 24.94^a^	E	4.92 ± 0.02^a^	G	63.33 ± 7.57^a^	G
	S2	163.33 ± 20.81^a^	E	5.75 ± 0.84^ab^	F	37.33 ± 4.50^b^	M
	S3	124.66 ± 27.15^b^	E	7.41 ± 0.09^b^	VP	31.0 ± 2.64^c^	M
	S4	325.0 ± 21.79^c^	E	6.33 ± 0.37^ab^	FP	40.0 ± 5.19^d^	M
Autumn	S1	102.66 ± 11.01^a^	E	4.57 ± 0.21^a^	G	61.0 ± 6.55^a^	G
	S2	105.0 ± 11.78^a^	E	6.39 ± 0.04^b^	FP	23.06 ± 9.01^b^	VP
	S3	28.33 ± 7.63^b^	G	7.63 ± 0.20^c^	VP	20.0 ± 0.0^c^	VP
	S4	45.00 ± 6.24^c^	E	5.77 ± 0.15^d^	FP	36.0 ± 2.0^d^	VP
Winter	S1	85.33 ± 13.05^a^	E	4.53 ± 0.35^a^	G	61.0 ± 3.46^a^	G
	S2	80.33 ± 12.66^b^	E	6.09 ± 0.54^ab^	FP	32.0 ± 4.0^b^	M
	S3	25.00 ± 5.00^c^	G	7.56 ± 0.05^b^	P	20.0 ± 3.46^c^	VP
	S4	50.00 ± 13.00^d^	E	6.29 ± 0.08^ab^	VP	48.66 ± 3.2^d^	M
Spring	S1	212.66 ± 33.26^a^	E	4.57 ± 0.21^a^	G	44.66 ± 3.05^a^	M
	S2	150.33 ± 3.78^b^	E	5.24 ± 0.10^b^	F	32.0 ± 1.15^b^	M
	S3	28.33 ± 10.40^c^	G	6.77 ± 0.02^c^	P	19.0 ± 3.46^c^	VP
	S4	92.00 ± 8.71^d^	E	5.83 ± 0.03^d^	FP	36.0 ± 1.52^d^	M

Abbreviations: BMWP, Biological Monitoring Working Party; E, excellent; EPT, Ephemeroptera, Plecoptera, and Trichoptera; F, fair; FP, fairly pollution; G, good; HFBI, Hilsenhoff Family Biotic Index; P, poor; VP, very pollution.

## 4 DISCUSSION

Freshwater ecosystems are threatened by anthropogenic activities, especially urbanization. Urbanization exerts a destructive impact on natural water systems by changing their morphology, hydrology, water chemistry, and biota (Hamid et al., 2019; Wallace et al., 2013). Biological indices are commonly used for assessing stresses caused by increased concentrations of nutrients or organic matter (Friberg, 2014; Kaboré et al., 2022). However, studies show that some water pollutants such as heavy metals, and agricultural products e.g., biocides can dramatically alter the structure and physiology of benthic organisms (Minelgaite et al., 2020; Ribeiro et al., 2013).

Today, biotic indices are used in many countries such as the UK (Medupin, 2019), China (Dou et al., 2022; Wu et al., 2020), Nigeria (Arimoro & Keke, 2017), France (Carayon et al., 2020), and Iran (Asadi Sharif et al., 2020) to assess the water quality. In particular, the assessment of the point and non‐point source pollution and its simultaneous impact on the water body, sediment, and ultimately the biota is rarely done. Based on the results of physical–chemical variables, S3 was identified as the most polluted station. This station has been affected by point source pollution wherein the high BOD level of 18 mg/L in the summer could exert catastrophic effects on the biota. The results of this study on the effect of pollution on the diversity of macro‐invertebrate communities were consistent with the results of the study conducted by Dou et al. (2022) in Lianhuan Lake. In both studies, the environmental variables such as DO, pH, ${\mathrm{NH}}_{4}^{+}$, ${\mathrm{NO}}_{3}^{-}$, and ${\mathrm{PO}}_{4}^{3-}$ caused a considerable reduction in the abundance of the sensitive macro‐invertebrate. In the present study, the results obtained for the mean concentrations of DO, BOD, ${\mathrm{NO}}_{3}^{-}$, and ${\mathrm{PO}}_{4}^{3-}$ indicated that the S3 health status was greatly affected by the point source. Considering the fact that macro‐invertebrates are a bridge between the ecosystem and physicochemical parameters, that could be assumed that the population structure was also altered in S3. The studies on the macro‐invertebrate fauna revealed an alteration from Baetidae and Caenidae in S2 to Chironomidae and Simuliidae in S3. The results evidently demonstrated that the resistant families have taken the place of the sensitive families as a result of point source pollution. Literature is replete with the reduction in the diversity and abundance of sensitive taxa such as Ephemeroptera, Plecoptera, and Trichoptera under contamination stress. Meantime, the diversity and abundance of tolerant taxa, such as Diptera (Chironomidae) and Oligochaeta tend to increase at contaminated sites (Odume et al., 2012; Rosa et al., 2014).

The results of the BIO‐ENV analysis exposed the major influence of physicochemical parameters on the distribution of macro‐invertebrates. But the fundamental question is, do heavy metals in water or sediment have the most influential impact on the arrangement of macro‐invertebrates? As per the results, increasing the concentration of heavy metals in sediment had a greater impact than that of the water concentration on the distribution of biota. Mrozinska and Bakowska (Mrozińska & Bąkowska, 2020) evaluated the impact of physicochemical parameters, sediment, and water‐heavy metals on the distribution of macro‐invertebrates. Their results showed that the distribution of macro‐invertebrates' communities was affected most in the order of physicochemical parameters > sediment heavy metals > water heavy metals, which was in line with our results.

As per Simper's statistical analysis, Chironomidae and Simuliidae had the most dissimilarity, notably in S3. The major difference between S3 and other stations was the point source pollution, which led to a transformation in the fauna and community structure of macro‐invertebrates from Beatidae and Caenidae to Chironomidae and Simuliidae. In the study by De Santiago et al. (de Santiago et al., 2020), the macrobenthic communities were examined in response to point sources of pollution in the subtropical regions of the Gulf of Oso. As per their results, the increase in the sewage discharge into the western Gulf, a distinct change in the community structure was raised, and the Ostracoda and oligochaete classes were dominated in the area. Similarly, Marchamalo et al. (2018) investigated the response of benthic invertebrates to human pressure in a volcanic basin at Birrís River. The results showed that the Chironomidae and Simuliidae families (Diptera order) and Baetidae family (Ephemeroptera order) predominated in the region throughout the year. The results indicated that in the areas with moderate pollution and low altitude: the Diptera order, and in the areas with high altitudes, severe organic pollution, and suitable substrates (sand, silt derived from volcanic ash): the Oligochaeta order dominated. This was consistent with our results about the presence of the Diptera in pollution sites.

With reference to the CCA statistical analysis, among the biotic indicators, the EPT was positively correlated with TEM, DO, and pH and negatively with HM. Moreover, the EPT index showed a decreasing trend with increased organic pollution in S3 and an increasing trend with decreased pollution in S4. According to Wright and Ryan (2016), the EPT index was inadequate to measure the effects of heavy metals independently because some families (i.e., Hydropsychidae) in the taxa of the EPT index are resistant to toxic metals and various types of organic pollution. As reported, macro‐invertebrates respond differently to heavy metals. However, sediments act as better heavy metal accumulators and serve as a more suitable predictor for metrics analysis (Ouma et al., 2022). Similar results were also observed in the present work wherein the sediment heavy metals exhibited higher effects on the distribution of macro‐invertebrates in the studied sites. In contrast, the HFBI and BMWP biotic indices revealed the “good” class water quality for S1 and the “poor” class water quality for S3.

Literature is fraught with studies on the effect of point source pollution on water quality status (Asadi Sharif et al., 2020; Malvandi et al., 2021). In the study of Asadi‐Sharif and Imanpur (Asadisharif & Imanpure, 2020), the effect of the point source pollution was assessed on the quality of the Disam River. As per the results of HFBI indices, the health status of the river was destroyed at the station with point source pollution. In the present study, the water quality was established in the “excellent” class as per the EPT index, which was not consistent with the BMWP and HFBI results. This could be associated with a large number of hydropsychidae families in the river, which are resistant to organic pollution and heavy metals and could cause errors in the EPT index assessment. Despite the most families included in the EPT index, the Hydropsychidae family is resistant to organic pollutants and heavy metals, yet BMWP and HFBI indices classified the S3 water quality in the “poor” category.

The trend in diversity indices is presented in Figure 4. The Shannon‐Wiener index is one of the most famous biodiversity indices, which was introduced in 1948 and comprised four classes based on the level of pollution and diversity (Shannon, 1948). The levels are as follows: (i) low diversity—high pollution, scored ≤1; (ii) low diversity—moderate pollution, scored 1 ≤ *x* ≤ 2; (iii) moderate diversity—low pollution, 2 ≤ *x* ≤ 3; (iv) high diversity—slight pollution, scored 3 ≤ *x* ≤ 4. The results of this study showed that the diversity indices of Shannon‐Wiener and Simpson indicated a decreasing trend in S3 in most seasons. On the contrary, the Dominance index showed an increasing trend in S3, which is in line with the results of Asadi Sharif et al. (2020). The major cause of the reduction in the rate of the diversity indices in S3 could have resulted from the point source pollution. Nonetheless, it should be noted that the reduction of diversity values cannot always result from pollution. Protasov et al. (2019) reported a decrease in the Shannon‐Wiener index with either decrease or increase in the Saprobity. Hence, it can be argued that the use of one index (diversity or biotic index) may not give a comprehensive view of an ecosystem condition. Therefore, simultaneous applications of various indices are recommended for the assessment of the health status of a river. The same conclusion was drawn by Morris et al. (2014) while seeking the best diversity index for the evaluation of the health status of German ecosystems.

## 5 CONCLUSION

The ecological quality of the Ojarud River, Ardabil, Iran, was assessed based on the macrobenthic communities in the selected stations. The findings established the highest frequent families from the Diptera order (Chironomidae and Simuliidae) and the Ephemeroptera order (Caenidae and Baetidae) while Pulmonata, Lepidoptera, and Coleoptera were among the least frequent orders. As per the seasonal study, the Ephemeroptera order showed the highest frequency at S2 in the autumn and Diptera was the most abundant order at S3 in summer. Moreover, the highest biomass of macro‐invertebrates was recorded in the summer in station 4 due to the dominance of the Gomphidae family, which are among the largest and heaviest macroinvertebrates. In contrast, the highest BOD and the least DO were detected in S3 influenced by the wastewater treatment plant prior to the station and decreased water flow. The average concentrations of Cd and Pb were, respectively, 0.059 and 1.392 mg/kg in Simuliidae and 0.031 and 0.773 mg/kg in Gamphidae and were found significantly higher downstream than headstream in both water and sediment. The BIO‐ENV statistical analysis showed that the physicochemical parameters (52%) were the most influential factors in the distribution of macro‐invertebrates followed by sediment heavy metals (26%), and water heavy metals (10%). Besides, S4 showed the least similarity (23%) to other stations in three seasons (spring, summer, and autumn) as per the Bray–Curtis similarity index analysis. Above all, the water quality of the Ojarud River was scored in the “excellent” class based on the EPT index. Nonetheless, the BMWP and HFBI indices placed the water quality in the “good” class at S1 and the “poor” class at S3. The results of this study exposed a sizable alteration in the diversity and abundance of macro‐invertebrate from the sensitive families to resistant and tolerant taxa under the contamination stress resulting from the point source pollution. The results of this study, yet again, verified the simultaneous applications of various indices for a more effective assessment of the health of a stream.

## AUTHOR CONTRIBUTIONS

**Aydin Mobasher:** Investigation (lead). **Abolfazl Bayrami:** Conceptualization (lead); funding acquisition (lead); methodology (equal); project administration (lead); resources (lead); supervision (lead). **Ehsan Asadi‐Sharif:** Formal analysis (equal); methodology (equal); software (lead); supervision (supporting); writing – original draft (equal). **Shima Rahim Pouran:** Methodology (supporting); supervision (supporting); visualization (lead); writing – review and editing (lead).

## CONFLICT OF INTEREST STATEMENT

The authors declare no conflict of interest.

## Supporting information


Figures S1–S2.

## Data Availability

The data of the present study are archived in Dryad data repository and are publicly accessible. Dryad dataset doi: 10.5061/dryad.18931zd2r.
