# Supplementary material for: Ecological indicators for qualitative assessment of Ojarud River: A case study
Source: Ecol Evol. 2023 Jul 17;13(7):e10310. doi: 10.1002/ece3.10310 (PMC10350820; doi:10.1002/ece3.10310)
Supplement: Supplementary file 1 — Figures S1–S2. [file ECE3-13-e10310-s001.docx]

Supporting Information

**Ecological indicators for qualitative assessment of Ojarud River: A case study**

Aydin Mobasher^1^, Abolfazl Bayrami^1*^, Ehsan Asadi-sharif ^2^, Shima Rahim Pouran^3*^

^1^ Department of Biology, Faculty of Science, University of Mohaghegh Ardabili, Ardabil, Iran.

[mobasheraydin9@gmail.com](mailto:mobasheraydin9@gmail.com)

[abolfazlbayrami@gmail.com](mailto:abolfazlbayrami@gmail.com) 0000-0001-7002-8605

^2^ Department of Soil and Water Research, Gilan Agricultural and Natural Resources Research and Education Center, AREEO, Rasht, Iran. [ehsanasadisharif@gmail.com](mailto:ehsanasadisharif@gmail.com)

^3^ Department of Environmental and Occupational Health, Social Determinants of Health Research Center, Ardabil University of Medical Sciences, Ardabil, Iran.

[rahimpooran@yahoo.com](mailto:rahimpooran@yahoo.com) 0000-0002-1891-5204

* Corresponding author:

Tel.: +98(45)33514702; Fax: +98(45)33514701; Email: abolfazlbayrami@gmail.com a_bayrami@uma.ac.ir

* Corresponding author (communicator):

Tel: + 98(45)33534757; Fax: +98(45)33514701; E-mail: rahimpooran@yahoo.com

sh.rahimpooran@arums.ac.ir


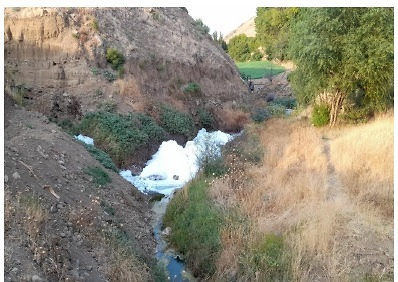


**Fig. S1** Sewage effluent combined with a lot of foam in the third station.

**Fig. S2** Images of a number of identified families.
